# MicroRNAs in Preeclampsia: An Overview of Biomarkers and Potential Therapeutic Targets

**DOI:** 10.3390/ijms26125607

**Published:** 2025-06-11

**Authors:** Mihaela Oancea, Dan Mihu, Cornelia Braicu, Ekaterina Isachesku, Ionel-Daniel Nati, Dan Boitor-Borza, Doru Mihai Diculescu, Stefan Strilciuc, Adrian Pană

**Affiliations:** 1 2nd Department of Obstetrics and Ginecology, “Iuliu Hațieganu” University of Medicine and Pharmacy, 400610 Cluj-Napoca, Romania; mihaelaoancea321@yahoo.com (M.O.); nati.ionel@yahoo.com (I.-D.N.); ddiculescu@yahoo.com (D.M.D.); 2 Department of Genomics, MEDFUTURE Institute for Biomedical Research, “Iuliu Hațieganu” University of Medicine and Pharmacy, 400610 Cluj-Napoca, Romania; ekaterina.isachesku@umfcluj.ro (E.I.); strilciuc.stefan@umfcluj.ro (S.S.); 3 Obstetrics and Gynecology I, Mother and Child Department, “Iuliu Hațieganu” University of Medicine and Pharmacy, 400012 Cluj-Napoca, Romania; danboitor@yahoo.com; 4 Center for Health Outcomes & Evaluation, Splaiul Unirii 45, 030126 Bucharest, Romania; adrian.pana@me.com

**Keywords:** miRNA, biomarker, therapeutic target, preeclampsia

## Abstract

Preeclampsia (PE) remains a significant obstetric challenge, having complex pathophysiology and limited early diagnostic and therapeutic options. MicroRNAs (miRNAs) have emerged as critical regulators in PE, offering insight into the molecular mechanisms underlying placental dysfunction and impaired maternal adaptation. Differentially expressed miRNAs in both placental tissue and maternal circulation, such as miR-155, play key roles in regulating angiogenesis, trophoblast invasion, and inflammatory pathways, all of which are central to the development of PE. Ongoing investigations increasingly highlight miRNAs as promising non-invasive molecular indicators for the early diagnosis and risk stratification of PE. Furthermore, therapeutic strategies targeting miRNA pathways using mimics or inhibitors show promise in correcting molecular dysfunctions and improving maternal and fetal outcomes. However, clinical translation faces several challenges, including targeted delivery, off-target effects, and the assessment of long-term efficacy. Overall, miRNAs hold significant potential as both diagnostic tools and therapeutic agents, marking a promising direction for improving care in PE pregnancies.

## 1. Introduction

Preeclampsia (PE) complicates roughly 1–8% of pregnancies worldwide and remains a leading cause of maternal and neonatal illness and death. Although clinical signs, such as elevated blood pressure and proteinuria, typically become apparent after 20 weeks of gestation (and can even appear postpartum), the pathological processes originate in the first trimester [1]. PE is a complex pregnancy complication characterized by systemic endothelial dysfunction and multi-organ involvement, typically emerging in the second half of pregnancy [1,2]. If untreated, it can evolve into eclampsia, HELLP syndrome (hemolytic anemia, elevated hepatic enzymes, low platelets), pulmonary edema, or placental abruption [2]. Fetal and neonatal complications include intrauterine growth restriction, medically indicated preterm delivery, stillbirth, and increased neonatal intensive care admissions [3]. Long-term follow-up studies also link PE with elevated cardiovascular risk in both the mother and child. Compared to normotensive pregnancies, preeclamptic gestations have higher rates of cesarean section and obstetric interventions.

Aberrant vascular adaptations during placentation in PE contribute to widespread endothelial dysfunction, diminished placental blood flow, and systemic inflammation [3]. These vascular irregularities result in impaired blood flow to the placenta, causing hypoxia and oxidative stress, which contribute to the clinical manifestations of the disease, such as hypertension and proteinuria [4]. Despite advances in biomarker research, including the evaluation of C-reactive protein, cytokines (IL-6, IL-8, TNF-α), oxidative stress markers, and genetic polymorphisms, few biomarkers have demonstrated consistent predictive accuracy for PE. Although prognostic research has explored biomarkers such as C-reactive protein, pro-inflammatory cytokines, oxidative stress indicators, and gene variants, reliable predictive markers remain limited in clinical applicability [5]. This highlights the urgent need to identify novel biomarkers and therapeutic targets to improve the management of PE [6]. Recent attention has focused on transcriptomic alterations, particularly the role of noncoding RNAs (ncRNAs), as promising candidates for managing this disease [7,8].

MicroRNAs (miRNAs) are small ncRNA transcripts, typically around 22 nucleotides in length [9]; miRNAs act as fine-tune regulators of gene expression [10]. A single miRNA can simultaneously target multiple mRNAs, while an mRNA can be targeted by multiple miRNAs, allowing for them to function as master regulators in complex molecular networks [9,11]. They exert their regulatory influence by binding to complementary sequences in target mRNAs, primarily in the 3′ untranslated regions (3′ UTRs). This interaction leads to either the suppression of translation or the degradation of the target mRNA, depending on the degree of complementarity between the miRNA and its target [9,10,11]. The impact of these miRNAs is primarily influenced by the identity of their target mRNAs and the critical cellular pathways they modulate [12].

miRNAs are highly stable even under challenging conditions, such as variations in pH, temperature, and enzymatic degradation, making them promising candidates as biomarkers from multiple biological matrices, including tissue, serum, plasma, urine, and saliva, which provide critical insights into placental function and disease pathophysiology [11,12,13].

In this study, we investigate the role of miRNAs in PE as potential early biomarkers for disease prediction and diagnosis. By presenting specific miRNAs associated with placental dysfunction, endothelial impairment, and systemic inflammation, this research aims to deepen our understanding of PE pathophysiology and assess its potential in enhancing early detection and guiding targeted therapeutic strategies.

## 2. Altered miRNA Patterns in PE

Throughout pregnancy, miRNAs are synthesized and secreted by various placental cells, including trophoblasts, contributing to the regulation of maternal–fetal communication and placental function. The placenta expresses over 500 miRNAs, many of which exhibit distinct expression patterns throughout pregnancy [14]. Several comprehensive analyses of altered miRNA signatures in PE reveal significant differences in miRNA expression profiles between PE patients and control pregnancies across various biological samples and methodologies [15,16,17,18,19]. Table 1 summarizes the findings from several studies that have evaluated the altered miRNA profile in PE compared to control pregnancies. For each study, the table details the patient cohort (including sample sizes and types, such as placenta or plasma), the evaluation method, and the specific signature altered in PE.

## 3. Altered miRNA in the Placenta

**Figure 1:**
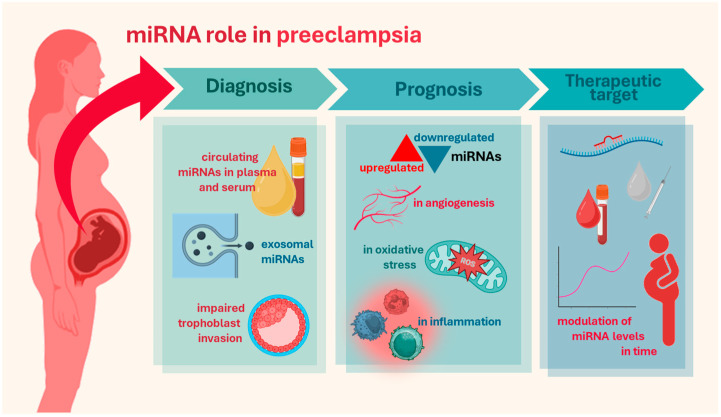
Overview of miRNA role in PE. miRNAs play a crucial role in the diagnosis of PE, as non-invasive biomarkers detectable in maternal blood, exosomes, and placental tissue. In prognosis, several miRNA were correlated with disease severity and progression, influencing key pathways such as angiogenesis, inflammation, and oxidative stress. Lastly, miRNAs can be considered a therapeutic target of PE by tracking their expression levels over pregnancy stages, providing insights into disease progression and treatment efficacy. Longitudinal miRNA analysis using liquid biopsy techniques enables non-invasive surveillance, supporting early intervention strategies to mitigate adverse outcomes. Figure created in BioRender.com.

The placenta, comprising both maternal and fetal components, is a primary source of circulating miRNAs during pregnancy, playing a crucial role in regulating maternal-fetal interactions and pregnancy outcomes. Figure 1 presents a schematic overview of the role of miRNAs in PE, highlighting their utility across diagnosis, prognosis, and therapeutic intervention. These placental-derived transcripts reflect local placental dysfunction, providing systemic insights into disease progression. Several differential expressions of placental miRNAs were correlated with the severity of PE, offering both prognostic and diagnostic value [11,20,21,22,23]. Most studies investigating these miRNAs in PE have focused on their role in endothelial dysfunction, highlighting their involvement in impaired angiogenesis, vascular inflammation, and genes related to oxidative stress, as shown in Table 2. The limitation of the data presented in Table 2 relates to the lack of precise information on the exact gestational week at sample collection.

miR-155 is one of the most extensively studied transcripts in the context of PE, with accumulating evidence supporting its involvement in the pathogenesis of the disorder. PE patients exhibit overexpression of miR-155 in both placental tissue and circulation [24]; many of these studies present miR-155 as a biomarker or therapeutic target [25,26,27,28]. Another study revealed that the upregulation of miR-155-5p in PE placentas suggests its potential role in influencing endothelial nitric oxide synthase (eNOS) expression [22]. These findings highlight miR-155-5p as a potential contributor to the pathogenesis of PE, underscoring its promise as a biomarker for this condition.

Elevated levels of miR-17, miR-20a, and miR-20b in PE suggest their involvement in early placental development through the modulation of genes such as *EPHB4* and *ephrin-B2* in both trophoblast and endothelial cells, suggesting their role in early placental formation [15]. In healthy placental tissue, miR-20b was detected in the syncytium and some villous mesoblasts, but was absent in capillary endothelial cells. In PE placentas, the overexpression of this transcript was observed, primarily in the syncytium, as revealed with in situ hybridization quantification [15].

In severe cases of PE, inverse expression patterns between miR-181a-5p and *MMP-9* have been observed, with correlations to lower gestational age at delivery and reduced neonatal birth weight, indicating their potential role in disease severity [29].

miR-454 plays a crucial role in regulating multiple cellular functions within trophoblast cells, influencing the key processes essential to proper placental development and function [30]. Elevated levels of miR-495 are associated with the progression of PE, suggesting a potential role for this miRNA in the disorder’s pathophysiology. miR-296 shows promise as a diagnostic biomarker for PE, with its altered expression linked to impaired trophoblast invasion and endothelial dysfunction. Its early detection in maternal plasma could aid in identifying at-risk mothers and improving pregnancy outcomes [31]. Another critical regulator, Decorin (DCN), inhibits trophoblast functions and induces the upregulation of miR-512-3p, which is also found to be elevated in PE placentas. miR-512-3p disrupts trophoblast migration, invasion, and differentiation, while paradoxically increasing PPP3R1 by suppressing USF2. These findings link miR-512-3p to the pathogenesis of PE and its potential as a biomarker [32].

Reduced expression of miR-3935 in both placenta and circulation has been linked to impaired epithelial–mesenchymal transition (EMT) processes in trophoblasts. It directly targets TRAF6, a key regulator that inhibits *RGS2*, thereby facilitating *ALKBH1*-induced demethylation of the *CDH1* promoter. This process leads to increased E-cadherin levels, which disrupts trophoblast EMT and contributes to defective placental development, ultimately playing a significant role in the pathophysiology of PE [33].

**Table 2:** Altered miRNAs in the placenta related to PE.

Type of Sample	Cohort	miRNA	Target Gene	Observation	Reference
Placenta	PE, healthy pregnancies	↑miR-17, -miR-20a, miR-20b	↓*ephrin-B2*	Angiogenesis	[15]
Placenta	PE (*n* = 12), healthy pregnancies (*n* = 12)	↓miR-126	↑*PIK3R2*	Diagnostic biomarker	[34]
Placenta	PE (*n* = 20) healthy pregnancies (*n* = 20)	↑miR-155	↓*FOXO3*	Inflammatory pathogenesis of PE	[35]
Placenta	PE (*n* = 20), healthy pregnancies (*n* = 20)	↑miR-155	↓*CYR61*	Pathogenesis of PE	[20]
Placenta	PE (*n* = 59) healthy pregnancies (*n* = 40)	↑miR-155	-	Pathogenesis of PE; prognostic biomarker	[22]
Placenta	Sever PE (*n* = 20), healthy pregnancies (*n* = 20)	↑miR-181	-	Trophoblast dysfunction, PE pathogenesis	[36]
Placenta	PE (*n* = 30), healthy pregnancies (*n* = 30)	↑miR-181a-5p	↓*MMP-9*	Severe PE correlated with adverse outcomes	[29]
Placenta	Sever PE (*n* = 30), healthy pregnancies (*n* = 30)	↓miR-424	-	Prognostic biomarker associated with severe PE	[37]
Placenta	PE (*n* = 20), healthy pregnancies (*n* = 20)	↓miR-454	↑*EPHB4*	Regulating trophoblast cell proliferation, apoptosis, and invasion function	[30]
Placenta	PE (*n* = 20), healthy pregnancies (*n* = 20)	↑miR-494	↓*SIRT1*	Senescence	[38]
Placenta	PE (*n* = 5), healthy pregnancies (*n* = 5)	↑miR-512-3p	↓*USF2*/*PPP3R*, *VEGF*	Extravillous trophoblast functions	[32]
Placenta	PE (*n* = 31), healthy pregnancies (*n* = 28)	↓miR-325	-	Pathogenesis of PE	[39]
Placenta and PBMC	-	↑miR-153-3p	↓*HMOX1*	Diagnostic potential	[40]
Placenta and serum	PE (*n* = 20), healthy pregnancies (*n* = 20)	↑miR-16	↓*VEGFA*	Diagnostic biomarker for severe PE	[41]
Placenta and serum	PE (*n* = 175) compared to control group (*n* = 350),	↑miR-155	-	Correlated with severe clinical features	[24]
Placenta and serum	PE (*n* = 200) healthy pregnancies (*n* = 50)	↑miR-296	-	Diagnostic biomarker for PE	[31]
Placenta and serum	PE (*n* = 15), healthy pregnancies (*n* = 15)	↓miR-3935	*TRAF6*/*RGS2*	Prognostic biomarker and therapeutic target of EMT signaling	[33]
Placental and myometrium tissue cohort	PE (*n* = 19), healthy pregnancies (*n* = 38)	↑miR-206	↓IGF-1	Prognostic biomarker	[21]
Umbilical cord tissues and primary trophoblast cells	PE (*n* = 68), healthy pregnancies (*n* = 30)	↑miR-495	↓HDAC2	Accelerates cell proliferation, invasion, and migration, but reduces apoptosis via P53/PUMA	[42]

↓ downregulation, ↑ overexpression.

## 4. Circulating miRNA in PE

The detection of circulating miRNAs, originating from various body tissues, including the placenta (Table 3), in biological fluids (such as serum, plasma, or urine) presents a valuable opportunity for real-time tracking diseases like PE from their onset through progression [43].

Sequencing approaches of blood and placental samples from three groups—early-onset PE (EOPE), late-onset PE (LOPE), and normal pregnancies—revealed that eight miRNAs were consistently identified across all groups, indicating their ubiquitous presence [44]. In contrast, other profiling studies revealed that miRNAs display specificity, being specific to PE subtypes. The analysis identified 492 target genes associated with these miRNAs, forming intricate interaction networks and highlighting several central genes with pivotal roles in the molecular landscape of PE [44].

A bioinformatics approach was employed to investigate the regulatory interactions between long noncoding RNAs (lncRNAs), miRNAs, and their associated target genes. In patients with PE, serum levels of H19, NEAT1, and SLC3A1 mRNA were found to be decreased, whereas miR-29b was significantly upregulated. No significant difference was observed in TUG1 expression when compared to healthy pregnancy controls. In early-onset and late-onset PE (EOPE and LOPE), serum H19 and TUG1 levels demonstrated inverse correlations with albuminuria, respectively. Additionally, NEAT1 and SLC3A1 levels were associated with ultrasound parameters in EOPE, while TUG1, miR-29b, and SLC3A1 exhibited significant correlations with ultrasound findings in LOPE [45].

Serum levels of miR-17, miR-363, and MALAT-1 have been proposed as potential diagnostic biomarkers for PE. miR-363 may be associated explicitly with early-onset cases, whereas reduced expression of MALAT-1 correlates with increased disease severity [46].

Notably, many studies included miR-155-5p as a biomarker for PE [14,20,22,26,35]. Deconvolution analyses further confirmed that several miRNAs are placenta-specific, underscoring their potential relevance in pre-eclampsia pathogenesis and biomarker development [25]. Studies have shown elevated levels of miR-155 and *CYR61*, as well as an increased *CYR61*/miR-155 ratio, in individuals with PE compared to healthy pregnancies. Furthermore, both serum *CYR61* concentrations and the *CYR61*/miR-155 ratio significantly differed between mild and severe forms of PE. These results indicate that miR-155, CYR61, and their ratio may serve as informative biomarkers for understanding the pathogenesis of PE and in assessing its severity [20].

In a prospective cohort of 15 women who subsequently developed PE and 29 gestational age-matched controls, plasma miR-125b was measured at 12–13 weeks of gestation. The analysis demonstrated a notable increase in miR-125b expression during early pregnancy (12th–13th gestational week) among those who subsequently developed the condition. Following delivery, miR-125b levels declined significantly, approaching baseline levels. These findings suggest that plasma miR-125b may serve as an early predictive biomarker for PE [16].

Significant differences in urinary miRNA profiles between PE and normal pregnancies suggest their potential for non-invasive disease monitoring [47]. In early gestation, differential expression of miR-184, miR-203a-3p, miR-205-5p, and miR-223-3p was reported, while hsa-miR-1-3p levels increased. Shifts in miRNA expression, including decreased miR-205-5p and miR-223-3p in the second trimester, suggest temporal variation. Additionally, miR-517 and miR-526 have shown diagnostic potential in predicting hypertensive disorders during early pregnancy [47,48].

**Table 3:** Altered circulating miRNA in PE.

Type of Samples and Cohort	miRNA	Target Gene	Observation	Reference
PE (*n* = 175), healthy pregnancies (*n* = 350)	↑miR-155	-	Prognostic biomarker	[24]
PE compared to a control group, blood samples	↑miR-155	↑*VPO1*, ↓*MOTS-c*	Markers of endothelial dysfunction	[49]
PE (*n* = 50), healthy pregnancies (*n* = 25), plasma	↑miR-155	-	CYR61/miR-155 Ratio as a biomarker for Diagnosis and severity of PE	[28]
PE (*n* = 15), healthy pregnancies (*n* = 29), plasma	↑miR-125b	↓*KCNA1* and *GPC1*	Inhibits cytotrophoblast invasion and impairs endothelial cell function; predictive marker and therapeutic target	[16]
PE (*n* = 30), healthy pregnancies (*n* = 30), plasma	↑miR-181a-5p	↓*MMP-9*	Associated with adverse outcomes in patients with severe PE	[29]
PE (*n* = 82) compared to healthy pregnancies (*n* = 78), serum	↑miR-29b	↓*SLC3A1*, ↑*TUG1*, ↑*H19*, and ↑*NEAT1*	Biomarker of PE severity	[45]
PE (*n* = 18), healthy pregnancies (*n* = 18), plasma	↑miR-206	-	Diagnostic biomarker	[21]
PE (*n* = 53) healthy pregnancies (*n* = 30), serum and blood	MiR-517 and miR-526	-	Prognostic biomarker	[48]
PE (*n* = 92) compared to healthy pregnancies (*n* = 78), serum	↓miR-363 and ↑miR-17	↓*MALAT1*	Biomarkers of PE risk, onset, and severity	[46]

↓ downregulation, ↑ overexpression.

## 5. Altered Exosomal miRNA in PE

Placental miRNAs are released into maternal circulation via microvesicles, exosomes, apoptotic bodies, or protein-bound complexes, making them detectable in maternal serum or plasma [50]. Identifying specific and reliable exosomal miRNA biomarkers could significantly enhance the management of PE (Table 4), particularly in severe and early-onset cases, by enabling earlier diagnosis and targeted monitoring, thereby reducing the risk of associated complications [50,51]. Recent research has identified six dysregulated miRNAs in PE exosomes, with the upregulation of miR-26a-5p, miR-152, and miR-155 and decreased levels of miR-18a and miR-221-3p compared to healthy pregnancies [52].

Exosomal miR-210 is actively secreted by trophoblast cells and may contribute to disease etiology through intercellular communication. Additionally, miR-210 bound to Ago proteins is passively released into the circulation, potentially as a byproduct involved in regulating cell death mechanisms, representing a possible consequence of the disease process [53].

Pathogenesis in PE is tightly linked to placental impairment and enhanced shedding of syncytiotrophoblast extracellular vesicles (STB-EVs) into the maternal circulation. An altered miRNA signature was identified in serum medium and large STB-EVs in the PE group versus healthy controls [54]. Additionally, there was an altered abundance of miR-9-5p in STB-EVs and serum levels [54]. Comparative analysis of exosomal miRNA expression revealed that miR-155 levels were markedly elevated in PE cases, but not in gestational hypertension, whereas miR-222 showed significant downregulation, specifically in PE. These differences in exosomal miR-155 and miR-222 expression between PE and GH suggest the presence of distinct pathological pathways underlying the two disorders [55].

**Table 4:** List of altered exosomal miRNA in PE.

Type of Samples	Cohort	Method for Evaluation	miRNA	Observation	Reference
Sera exosomes	PE (*n* = 10) and normal pregnant women (*n* = 10)		↑miR-26a-5p, miR-152, and miR-155; ↓miR-18a and miR-221-3p	Participates in the development and progression of PE by targeting trophoblast cells	[52]
Plasma exosomes and placenta	PE (*n* = 8) and normal pregnant women (*n* = 8) Placenta PE (*n* = 13) and normal pregnant women (*n* = 7)		↑miR-210	miR-210 is secreted from the trophoblast, regulated intercellular communication as Ago-bound miR-210	[53]
Gestational hypertension (GH) and PE exosomes and placentar exososmes	PE (*n* = 15) and GH (*n* = 15) and normal pregnant women (*n* = 15)	qRT-PCR	↑miR-155 and ↓miR-222 in PE but not in GH	miR-155 and miR-222 regulate different pathological pathways	[55]
Plasma exosomes	Profiling PE (*n* = 5) and normal pregnant women (*n* = 5)	RNAseq (Illumina NextSeq 2000 platform)	↑miR-122-5p, miR-4535-3p, miR-20a-5p, miR-302a-5p, miR-1-3p, miR-125b-2-3p, miR-144-5p ↓miR-144-3p, miR-143-3p, miR-183-5p, miR-185-5p, miR-186-5p, miR-501-3p, miR-30a-5p, miR-96-5p, miR-26b-5p	Not only suitable biomarker candidates, but also novel mechanistic insights related to PE	[50]
Syncytiotrophoblast extracellular vesicle release (STB-EV)	Profiling PE (*n* = 6) and normal pregnant women (*n* = 6)	RNAseq (Illumina NextSeq 2000 platform)	↑miR-4488, miR-3196, miR-4516, miR-193b-5p, miR-210-3p, miR-27a-5p, miR-3656, miR-113, miR-3960, miR-6089, miR-127-3p, miR-317, miR-338, let-7b-5p, miR-9-5p, miR-483-3p, miR-493-5p, miR-455-3p, miR-99a-5p, miR-370-3p, miR-10b-5p, let-7c-5p, miR-92b-3p ↓ miR-151a-5p, miR-26b-5p, miR-519d-3p, miR-877-5p, miR-421, miR-106b-5p, miR-93-5p, miR-222-3p, hsa-let-7e-5p, miR-374a-5p, miR-519b-3p, miR-30b-5p, miR-519c-3p, miR-194-5p, miR-221-3p, miR-652-3p, miR-324-5p	miR-9-5p as a potential biomarker	[54]

↓ downregulation, ↑ overexpression.

## 6. miRNAs as Therapeutic Targets in PE

**Figure 2:**
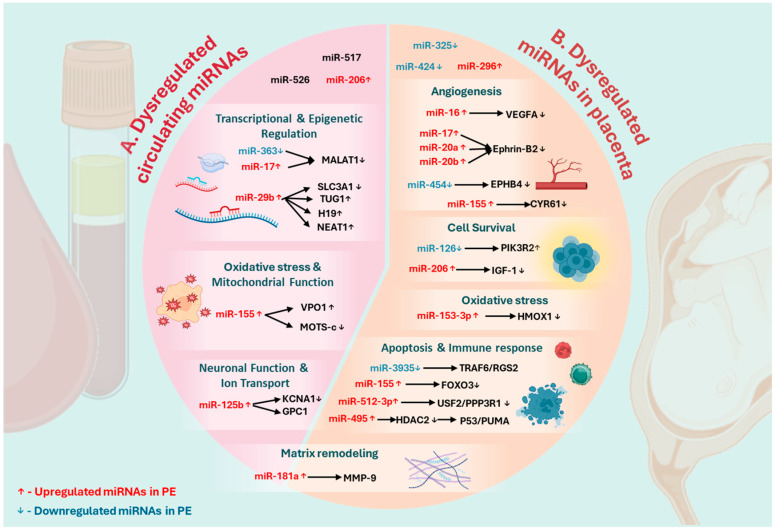
Dysregulated miRNAs in PE. PE is associated with the dysregulation of circulating and placental miRNAs. Since miRNAs exert their effects by targeting specific genes, their dysregulation leads to the altered expression of these target molecules, which, in turn, directly or indirectly perturb key biological processes within the cell. (**A**) Altered circulating miRNAs influence transcriptional and epigenetic regulation (e.g., miR-29b↑, miR-363↓), oxidative stress (e.g., miR-155↑), and neuronal function and ion transport (e.g., miR-125b↑). (**B**) Similarly, in the placenta, dysregulated miRNAs modulate angiogenesis (e.g., miR-16↑, miR-17↑, miR-20a↑, miR-20b↑), cell survival (e.g., miR-126↓, miR-206↑, miR-155↑), oxidative stress (e.g., miR-153-3p), apoptosis, and immune response (e.g., miR-512-3p↑, miR-495↑, miR-3935↓). The dysregulation of matrix remodeling (e.g., miR-181a↑) was observed in both placental and circulating miRNAs. This highlights the critical role of miRNA-mediated gene regulation in PE pathophysiology, suggesting that miRNAs may serve as potential biomarkers or therapeutic targets for managing the disease. Created in BioRender.com.

Therapeutic approaches utilizing miRNA mimics or inhibitors represent an innovative strategy to correct molecular dysfunctions [56], including those implicated in PE, as observed in Figure 2. Two primary approaches are under investigation: (1) miRNA mimics, which are synthetic oligonucleotides designed to restore the function of downregulated miRNAs and thereby normalize the expression of their target genes; and (2) antagomiRs, chemically modified inhibitors that specifically bind miRNAs that are aberrantly overexpressed [57,58]. A major challenge in developing miRNA-based therapies is achieving targeted delivery to the placenta or endothelial cells, which is essential to minimize off-target effects and enhance therapeutic efficacy [56,57,58]. To evaluate these therapeutic strategies, relevant in vitro and in vivo models of PE—such as trophoblast cell cultures and animal models exhibiting key features of the disease—are critical for assessing the safety, delivery efficiency, and functional outcomes of miRNA modulation [56,57].

Table 5 summarizes key studies that explore the association between altered miRNA expression and critical pathophysiological processes in PE, including impaired angiogenesis, trophoblast dysfunction, and systemic endothelial damage. Additionally, these studies investigate the therapeutic potential of modulating specific miRNA levels to directly target and correct the underlying molecular abnormalities of the disease.

In PE, miRNAs such as miR-155 are upregulated, contributing to pathogenesis by inhibiting pro-angiogenic or trophoblast-related genes [20,59]. In PE, miR-155 plays a crucial role in modulating cellular functions by regulating *PKG1*. Additionally, its expression is regulated by the NFκB signaling pathway [26]. miR-155 regulates the functions of trophoblast cells, including cell viability, apoptosis, mobility, and oxidative stress. By modulating these signaling pathways, miR-155 affects trophoblast survival, migration ability, and response to oxidative stress, which are crucial processes for normal placental development and function [35]. DEHP (Di-2-ethylhexyl phthalate)-induced miR-155-5p plays a regulatory role in trophoblastic lipid metabolism by suppressing the cAMP/PKA signaling pathway in human trophoblastic HTR-8/Svneo cells. This suggests that miR-155-5p acts as a mediator of DEHP’s effects on trophoblast function, highlighting its potential impact on placental development and associated disorders [59].

Let-7a was highly expressed in early-onset severe PE with low methylation of let-7a-3p. It inhibited cell viability and cycle progression while promoting apoptosis in JEG-3 cells by downregulating *Bcl-xl* and *YAP1* [60]. The demethylation of let-7a-3p further increased let-7a expression and apoptosis. In vivo, let-7a reduced tumorigenic potential and enhanced apoptosis, highlighting its role as a key regulator in PE progression [60].

miR-31-5p knockdown partially reverses the autophagy enhancement caused by SNHG5 silencing in trophoblast cells, suggesting a regulatory axis between SNHG5 and miR-31-5p [61], with potential implications in placental function and pregnancy disorders [61]. The overexpression of placental miR-513c-5p contributes to the development of PE by regulating trophoblast biological functions through the inhibition of LRP6 [62].

This disruption in endothelial function, a hallmark of PE, highlights the complex interplay between molecular changes and vascular homeostasis. Elevated levels of *VPO1* and miR-200c, coupled with reduced human and MOTS-c levels, are closely linked to the dysregulation of endoglin expression, further contributing to the endothelial dysfunction characteristic of PE [49]. MiR-126 enhances the functions of endothelial progenitor cells (EPCs), including proliferation, differentiation, and migration, by suppressing the antiangiogenic factor *PIK3R2*. Inhibition of miR-126 impairs the functionality of endothelial progenitor cells (EPCs), whereas their overexpression or downregulation of PIK3R2 improves it. In pregnant rats, miR-126 promoted vascular sprouting and increased placental and fetal weights, highlighting its critical role in angiogenesis and placental vasculogenesis, with potential as a therapeutic target for PE [34].

Under hypoxic conditions, the expression of miR-141 is significantly increased in HTR-8/SVneo cells, leading to enhanced apoptosis and reduced invasion and vascularization. It directly targets *CXCL12β*, inhibiting its translation, and modulates key molecules by downregulating *MMP2, p62*, and *LC3B* while upregulating *ROCK1* and *RhoA*. Arachidonic acid counteracts the effects of *CXCL12β* suppression, restoring invasion and decreasing apoptosis. By targeting the *CXCL12β* and *CXCR2*/*4* signaling pathways, hypoxia-induced miR-141 disrupts trophoblast function, potentially impairing placental development [63].

Alteration of the expression levels for miR-181a-5p has been identified as a significant factor in the regulation of trophoblast dysfunction. Overexpression of this transcript affects critical cellular processes in trophoblasts (cell proliferation, cell cycle progression, and invasion), which are essential for normal placental development. By inducing cell cycle arrest and apoptosis, this transcript disrupts the balance of trophoblast growth and renewal. Additionally, its suppression of invasive capacity hinders the ability of trophoblasts to effectively remodel maternal spiral arteries, a crucial step in establishing proper uteroplacental blood flow [36].

In severe PE, decidua-derived mesenchymal stem cells display heightened miR-16 expression, which markedly suppresses their proliferative capacity and disrupts both migration and cell-cycle dynamics. This also reduced DMSC-mediated support for blood vessel formation and trophoblast migration. *VEGF-A*, which is crucial for these processes, was found to be inversely correlated with miR-16 expression in DMSCs from patients with severe PE, suggesting that altered miR-16 levels in DMSCs may contribute to the pathogenesis of the disorder [41].

Circ_0001326 was found to regulate HTRA1 by sequestering miR-188-3p. In vitro, rescue assays show that the reintroduction of miR-188-3p fully reversed the impaired proliferative and migratory capacities observed upon circ_0001326 depletion. Furthermore, silencing HTRA1 diminished the phenotypic changes in trophoblast cells induced by the inhibition of miR-188-3p [64].

Overall, miRNA-based therapies hold great promise for correcting the molecular imbalances in PE. They offer the potential for highly specific gene-targeted interventions that address the root causes of the disorder, paving the way for innovative and effective treatments for this complex pregnancy complication.

**Table 5:** miRNAs as therapeutic targets in PE.

Testing System	miRNA	Target Gene	Observation	Reference
HUVEC, HTR-8/SVneo	miR-16	*VEGFA*	Cell proliferation, EMT, and angiogenesis	[41]
HTR-8/SVneo	miR-31-5p	*SNHG5*	Trophoblast autophagy	[61]
HTR-8/Svneo	miR-155-5p	*cAMP*/*PKA*	Regulated lipid metabolism by	[59]
HTR-8/SVneo, JEG-3, 293T	miR-155-5p	*FOXO3*	Cell viability, apoptosis, mobility, and oxidative stress	[35]
HTR-8/SVneo, BeWo, HEK-293T	miR-155-5p	*CYR61*	Trophoblast migration	[20]
HTR-8/SVneo, JAR c	miR-181-5p	*-*	Proliferation, cell cycle progression, apoptosis, and invasion	[36]
HTR-8/SVneo	miR-188-3p	*HTRA1*/Circ_0001326	Cell growth, invasion, migration, and EMT	[64]
JEG-3	let-7a-3p	*Bcl-xl* and *YAP1*	Regulates cell apoptosis in trophoblasts	[34]
HTR-8/SVneo	miR-141	*CXCL12β*/*CXCR2*/4	Regulates trophoblast apoptosis, invasion, and vascularization by blocking signal transduction	[63]
Primary cell culture placenta/cord blood	miR-126	PI3K–Akt signaling Axis	Cell proliferation and differentiation; colony Formation and migration	[34]
Trophoblast	miRNA-494	*SIRT1*, NLRP3, IL-1β	Senescence	[38]
HTR-8/SVneo	MiR-513c-5p	*LRP6*	Proliferation, invasion migration, and promoted apoptosis	[62]
PE mouse model	miR-155	*PKG1*	Cell invasion, migration, and apoptosis via NFκB pathway	[26]

## 7. Conclusions

Current evidence shows that miRNAs have emerged as pivotal players in the pathophysiology of PE, acting as biomarkers and therapeutic targets. Their differential expression in placental tissue and maternal circulation provides a molecular snapshot of the underlying disruptions in placental development and maternal adaptation to pregnancy. MiRNAs, such as miR-155, have been extensively studied, highlighting their regulatory roles in angiogenesis, trophoblast invasion, and inflammatory responses—processes that are highly relevant to PE pathogenesis. These findings underscore the potential of miRNAs as minimally invasive tools for the early diagnosis and stratification of disease severity.

From a therapeutic perspective, miRNA mimics and inhibitors hold promise for addressing the molecular dysfunctions in PE. The ability to selectively modulate miRNA activity could pave the way for innovative treatments aimed at restoring placental function and reducing the risk of complications for both mother and fetus. However, the clinical translation of miRNA-based therapies faces several challenges, including the development of efficient and safe delivery systems, minimizing off-target effects, and ensuring long-term efficacy.

Looking ahead, future research should prioritize the validation of miRNA-based biomarkers in large, diverse patient cohorts, with a focus on the gestational stage and the specific placental origin (maternal or fetal). Integrating multi-omics approaches—such as combining miRNA profiling with proteomics, transcriptomics, and metabolomics—will provide a more comprehensive understanding of the molecular mechanisms underlying PE. Additionally, biobanking early pregnancy samples is essential for conducting longitudinal studies that can track disease progression and identify early predictive markers. The application of artificial intelligence and machine learning tools to analyze complex miRNA datasets holds great promise for improving predictive modeling and risk stratification in PE pregnancies.

Advancements in delivery technologies, such as nanoparticle-based systems, could significantly enhance the feasibility of miRNA therapeutics by improving stability, enabling targeted delivery to placental tissue, and minimizing off-target effects—one of the major challenges currently limiting clinical translation.

In conclusion, while the field of miRNA research in PE is still evolving, miRNAs’ dual potential as biomarkers and therapeutic agents presents a transformative opportunity to enhance maternal and fetal outcomes. Close collaboration among scientists, obstetric clinicians, and biopharmaceutical developers is essential to translate these molecular discoveries into reliable diagnostics and effective treatments for this life-threatening obstetric disorder.

## Figures and Tables

**Table 1:** Altered miRNA pattern in PE.

Patient Cohort	Altered Signature	Reference
PE (*n* = 10) and control pregnancies (*n* = 10), placenta, microarray	↑miR-20b, miR-516a-5p, miR-512–3p, miR-2277, miR-524-3p; ↓miR-151-3p, miR-146a, miR-192, miR-34c-5p; validation by in situ hybridization of miR-17, miR-20a, and miR-20b	[15]
PE (*n* = 6) and control pregnancies (*n* = 6), plasma, miRNA microarray	↑ let-7a-5p, miR-15a-5p, miR-92a-1-3p, miR-106a, miR-125b, miR-130a-3p, miR-191-5p, miR-374a-5p, miR-574-5p; ↓miR-22–5p, miR-93-5p, miR-126-3p, miR-204-3p, miR-365a-3p, miR-559-5p, miR-4264-5p	[16]
PE (*n* = 20) and control pregnancies (*n* = 20), placenta miRNA microarray	↑miR-210; ↓miR-328, miR-584, miR-139-5p, miR-500, miR-1247, miR-34c-5p and miR-1	[17]
PE (*n* = 31) compared to healthy pregnancies (*n* = 32), 32 miRNA profiling using qRT-PCR, plasma	↑miR-210, miR-375, miR-197-3p, miR-132-3p, miR-29a-3p, miR-328, miR-24-3p, and miR-218-5p; ↓miR-302b-3p, miR-191-5p, and miR-17-5p	[18]
Early-onset PE (EOPE) GSE103542, GSE74341, and GSE44711	↑miR-1914, miR-431, miR-485-3p, miR-500b, miR-145*, miR-3941, miR-367*, miR-875-3p; ↓miR-542-3p, miR-2276-126*, miR-544b, miR-3652, miR-937, miR-3907, miR-3190, miR-4253, miR-1274a, miR-3942, miR-1471, miR-148b*, miR-218, miR-1537, miR-3943, miR-19a*, miR-3646, miR-302a, miR-30a	[19]

↓ downregulation, ↑ overexpression, * passenger strand of the transcript.
